# The Utilization of Formalin Fixed-Paraffin-Embedded Specimens in High Throughput Genomic Studies

**DOI:** 10.1155/2017/1926304

**Published:** 2017-01-26

**Authors:** Pan Zhang, Brian D. Lehmann, Yu Shyr, Yan Guo

**Affiliations:** ^1^Department of Cancer Biology, Vanderbilt University, Nashville, TN, USA; ^2^Department of Biochemistry, Vanderbilt University, Nashville, TN, USA; ^3^Department of Biostatistics, Vanderbilt University, Nashville, TN, USA

## Abstract

High throughput genomic assays empower us to study the entire human genome in short time with reasonable cost. Formalin fixed-paraffin-embedded (FFPE) tissue processing remains the most economical approach for longitudinal tissue specimen storage. Therefore, the ability to apply high throughput genomic applications to FFPE specimens can expand clinical assays and discovery. Many studies have measured the accuracy and repeatability of data generated from FFPE specimens using high throughput genomic assays. Together, these studies demonstrate feasibility and provide crucial guidance for future studies using FFPE specimens. Here, we summarize the findings of these studies and discuss the limitations of high throughput data generated from FFPE specimens across several platforms that include microarray, high throughput sequencing, and NanoString.

## 1. Introduction

The technique of FFPE is a widely used histological method that uses formalin to fix and paraffin embedding to preserve tissues for extended periods of time. However, the advantages of FFPE processing such as technical ease and low storage cost come at the expense of the sample quality. During the process of fixation, the tissue DNA can be altered by chemical modification, DNA trapping and fragmentation [1, 2], resulting from extensive cross-linking between proteins and nucleic acids [3].

The effects of formalin-fixation are far greater on RNA, as RNA can be altered by severe RNA degradation, chemical modification [4], poly-A tail damage [5], and covalent modification of RNA nucleotide bases by monomethylol (-CH2OH) addition [6]. These covalent modifications can impact reverse transcription from mRNA to cDNA and significantly alter gene expression profiling.

Despite these shortcomings, researchers have successfully been using RNA and DNA extracted from FFPE specimens for high throughput genomic studies. Herein, we review the applications of FFPE specimens in high throughput genomic studies using several technologies, including microarray, high throughput sequencing (HTS), and NanoString.

## 2. Technical Challenges and Concerns

One major challenge in using FFPE specimens in genomic assays is the low quality and quantity of nucleic acids extracted from FFPE blocks. The process of FFPE is designed to well preserve cellular proteins that can be evaluated by immunohistochemistry based assays rather than preserving DNA or RNA. It is known that formalin-fixation can cause nucleic acids fragmentation, degradation, and cross-linking to proteins [1–3, 7–11]. In addition, the long storage time can further compromise the quality of the nucleic acids within FFPE blocks [12]. Nucleic acid degradation and crosslinking to proteins can significantly reduce the quantity of DNA and RNA extracted from FFPE specimens, while nucleic acids fragmentation can reduce library fragment size and uniformity. Further complicating genomic assay is the limited quality control measurements that are performed on FFPE specimens such as traditional RNA integrity number (RIN) measurements that do not truly reflect the success chance of sequencing from FFPE specimens [13]. Together, reduced quantity and integrity of the extracted nucleic acids can lead to the failure of HTS library construction.

Prior to sequencing, commercially available kit, such as Illumina TruSeq, is required to assemble a sequencing library. Most commercial kits have similar performance. Unlike DNA, there are several methods to enrich for RNA prior to library construction that use depletion of highly abundant ribosomal RNA or oligo-dT to capture mRNAs with polyA tails. For RNA library construction, ribosomal RNA depletion method is preferred to oligo dT capture because many mRNA transcripts from FFPE specimens have lost their polyA tails from to extensive fragmentation [14]. Both Illumina and SOLiD HTS platforms have been demonstrated to work well with FFPE derived libraries and different platforms does not seem to have a bias toward FFPE specimens [15, 16]. While HTS libraries have been constructed from nucleic acids with poor quality, those studies [17, 18] have shown that the sequencing data generated were less than ideal quality.

## 3. Microarray

Gene expression microarray uses large-scale arrays of fluorescent oligonucleotide probes to measure mRNA expression across many genes simultaneously and was the driving force for high throughput gene expression studies prior to the introduction of RNA-seq. During the gene expression microarray era, FFPE specimens had been extensively used for expression profiling purposes [19–22]. Because the quality of RNA extract from FFPE specimens is always of questionable quality, many studies [23–28] were conducted to evaluate the integrity of FFPE gene expression microarray data by comparing the gene expression consistency between paired FFPE and fresh frozen (FF) samples. All of the comparative studies have found that reasonable consistency of gene expression quantified from FFPE and FF specimens likely attributed the oligonucleotide probes measure expression being located at several positions across a gene. In addition to mRNA transcript quantification, microarray technology has been adapted to measure DNA copy number, single nucleotide polymorphisms (SNPs), and DNA methylation.

The most frequent types of variation in the genome are single base differences between two DNA sequences and genotyping microarray has been developed to detect single nucleotide polymorphisms in genomic DNA. Although DNA is more stable than RNA, the quality of DNA extracted from FFPE specimens can be considerably compromised by artefactual nucleotide changes introduced by formalin-fixation. Therefore, many studies have evaluated the feasibility of using DNA extracted from FFPE specimens for genotyping array analysis [29–33]. These studies have shown a high concordance in SNP calls between FF and FFPE specimens. Encouraged by these findings, researchers have widely used FFPE specimens in a variety of genotyping array studies [30, 31, 34–37]. In addition to SNP detection, genotyping arrays can also be used to estimate DNA copy number variance (CNV). However, CNV estimation from DNA obtained from FFPE specimens can be challenging, as DNA usually degraded and fragmented. Nonetheless, several modified protocols have been reported and different array platforms have been tested for the practicability of performing CNV analysis with FFPE specimens [29, 33, 36]. All these studies show plausible concordance of CNVs identified between paired FFPE and FF specimens.

In addition to CNV estimation from genotype arrays, comparative genomic hybridization (CGH) arrays have been developed as a genome-wide, high-resolution technique for the detection of copy number variations between two genomes. As aforementioned, CNV detection is more susceptible to the fragmented nature of DNA extracted from FFPE specimens. One study has shown that FFPE specimens can have spurious copy number variation in array-CGH profiles [38]. For successful CNV estimation from array-CGH, several requirements for DNA have been suggested for FFPE [39]. First, it was found that only FFPE tissues that supported polymerase chain reaction (PCR) amplification of >300 bp DNA fragment provided high quality, reproducible array-CGH data. Second, roughly 10 ng DNA from FFPE tissues is needed as input for array-CGH analysis prior to whole genome amplification. Third, high tumor cellularity of greater than 70% tumor DNA was required for reliable array-CGH analysis [39].

Prior to hybridization, DNA must undergo whole genome DNA amplification and several amplification methods can also affect the quality of array-CGH data [40]. Random-primed amplification was found to be superior to degenerate oligonucleotide-primed amplification [40]. Several studies have proposed optimized protocols for array-CGH analysis using DNA from FFPE specimens [41, 42]. Comparison studies using either paired FF specimens or fluorescent in situ hybridization (FISH) methods as a gold standard have demonstrated that array-CGH are reliable for CNV estimation from FFPE specimens [43–45]. This reliability has allowed for a clinical application of array-CGH to distinguish Spitz nevus and melanoma in FFPE specimens [46].

DNA can be modified by several mechanisms that can alter gene transcription including methylation of CpG sites and microarray technologies have been adapted to measure global methylation patterns of DNA. These methods largely rely on bisulfite treatment to convert unmethylated cytosine to uracil and the latest methylation EPIC BeadChips from Illumina can interrogate over 850,000 CpG sites at single nucleotide resolution. Several studies compared methylation values measured from Illumina methylation arrays on paired FFPE and FF specimens and found high level of concordance (*R*^2^ > 0.95) [47–50]. While study did report lower concordance between FFPE and FF specimens (*r* = 0.6) [51], others have questioned the statistical considerations and batch effect that may have impacted this study [52]. The overall good performance of FFPE in methylation arrays is likely due to the better stability of DNA compared to RNA. To date, many epigenetic methylation studies have used FFPE specimens as their source [53, 54].

## 4. RNA-Seq

With the rise of HTS technology, RNA-seq has inevitably replaced microarray as the platform of choice for expression profiling technology [55–59]. RNA-seq provides numerous advantages over microarray technology, including the identification of all RNAs in the library rather than RNA with predesigned probes, allowing the expression quantification at multiple levels (gene, transcript, and exon) without designing specific probes and permitting the additional discovery opportunities such as gene fusion and allelic specific expression.

Similar to microarray technology, FF tissue samples provide the highest data quality. However, majority of specimens are processed by FFPE and researchers have been applying the same strategy as during the microarray era, evaluating the accuracy and repeatability of gene quantification using HTS technology by comparing matched pairs of FF and FFPE specimens from the same subject.

Norton et al. calculated the correlation of gene expression across nine matched pairs of FF and FFPE specimens and Pearson correlations ranged from 0.60 to 0.83 [60]. Graw et al. analyzed RNA-seq data from six pairs of FF/FFPE tumor samples and found that the correlations of gene expression data were greater than 0.89. The same study also reported 99.67% concordance between sequence variations identified from FFPE RNA and FF DNA [61]. In Hester et al.'s study, storage time was shown to impact concordance between paired FF and FFPE specimens with high concordance of specimens stored less than 2 years (*r*^2^ = 0.99) compared to FFPE specimens with storage greater than 20 years (*r*^2^ = 0.84) [62]. Hedegaard et al. compared the expression profiles from 27 FFPE and FF pairs from different tissues (colon, bladder, and prostate) and with different storage time. The results revealed a high degree of Pearson correlation (*r* > 0.90) across all pairs [18]. Zhao et al. used two ribosomal RNA removal kits (Ribo Zero and Duplex-Specific Nuclease) to sequence paired FFPE and FF specimens. Both protocols resulted in a Pearson correlation of about 0.90 between matched pair of FFPE and FF specimens [63]. Eikrem et al. compared the gene expression profiles across 16 pairs of FF and FFPE specimens and the correlation of the average expression is 0.97 [64]. Li et al. also reported a correlation more than 0.91 between FF and FFPE pairs [65]. These studies show that reliable gene expression data can be obtained from whole transcriptome sequencing of FFPE specimens; provided tissues blocks have not been stored from long periods.

In addition to gene expression quantification, RNA-seq data can be mined for single nucleotide variants and structural alterations such as gene rearrangements that result in hybrid transcripts [66]. However, unlike gene expression quantification, these additional data mining opportunities do not apply well for RNA-seq data generated from FFPE specimens. One comparative study found that only 24% of high-confidence fusion transcripts detected in FF specimens were also detected in matched FFPE specimens [60]. This low recovery rate occurs despite threefold increases sequencing depth. Another study found that between SNVs identified from RNA-seq replicates from FFPE specimens showed extremely poor genotype consistency (<50%), rendering it unreliable for SNV detection [14].

Thus far, overwhelming findings provided emerging evidence of the accurate expression profiles obtained from FFPE specimens; an increasing number of studies began to use RNA-seq technology on FFPE specimens to perform gene expression profiling [67–75]. While gene expression quantification has produced reliable results, other data mining opportunities such as gene fusion and SNV detection have been found to be not feasible with FFPE specimens.

## 5. Small RNA-Seq

MicroRNAs (miRNA) are small noncoding RNA molecules containing around 22 nucleotides and have been found to play an important role in many biological processes. MiRNAs function through base-pairing with complementary sequences within mRNA molecules and these mRNA molecules are subsequently silenced. HTS has also revolutionized the miRNA research area. Compared to traditional methods such as TaqMan gene expression assay and microarray, HTS enables the detection of almost all small RNAs present in the samples, including novel and underexpressed miRNAs as well as small RNAs of other categories [76].

Since miRNAs are more stable than RNA molecules [77–79], HTS is quite promising for quantifying miRNA profiles from FFPE specimens. Several pioneering studies using matched FF and FFPE specimens have already been performed to evaluate the usefulness of FFPE specimen for miRNA-seq technology. These studies have found that miRNA-seq data generated from FFPE specimens have similar number of total reads but tend to have a slightly shorter average read length after trimming for adapter sequences [80–83].

In addition, the proportion of reads that can be mapped to miRNAs was also lower in FFPE specimens [80, 81]. The decreased mapping could be due to small fragments of other RNA species such as degraded lncRNAs and mRNAs in the small RNA library [81]. Most studies agree that the small RNAs composition from FFPE specimens is similar to that from FF specimens [81, 83], and correlations between miRNA expression levels quantified from paired FF and FFPE specimens range from 0.71 to 0.98 [80, 81, 83]. More interestingly, against common intuition, two studies found that storage time of the FFPE blocks did not affect the quality of miRNA-seq data [81, 83]. These studies further showed that while the total miRNA expression profile is highly correlated between matched FF and FFPE specimens, the relative read count of each miRNA is dependent on GC content. Specifically, GC-poor miRNAs were shown to be more degraded than GC-rich miRNAs [80].

Encouraged by these validation studies, researchers began to apply HTS miRNA-seq to FFPE specimens [84, 85]. Plieskatt et al. applied miRNA-seq on FFPE preserved nasopharyngeal carcinoma tissues. They found that FFPE tissue can yield RNA of sufficient quality for downstream sequencing analysis. Using the miRNA profile generated from these FFPE specimens, the authors identified Epstein Barr Virus miRNAs as potential NPC biomarkers [84]. Riester et al. collected 16 osteosarcomas FFPE specimens and 14 osteoblastomas FFPE specimens. miRNA-seq analysis of these 30 FFPE specimens allows the authors to identify miR-210 as a discriminatory marker that distinguishes between osteoblastoma and osteosarcoma [85].

## 6. DNA-Seq

HTS technologies have been widely used to characterize variations and quantity of DNA from both normal and diseased tissue. DNA-sequencing can be used to characterize genomic variants such as SNV, insertions/deletions (Indels), copy number variations (CNVs), and structural gene rearrangements. HTS DNA-seq performs better with high quality DNA from FF specimens as starting materials. However, FFPE specimens have also been evaluated using DNA-seq.

Similar to comparisons of microarray and RNA-seq, many studies have used matched paired FFPE and FF specimens to evaluate the quality of genomic variants identified from FFPE specimens. The overall concordance of SNV calls between FF and FFPE specimens across different studies ranges from 70% to 99.8% [15–18, 86–92]. In most cases, more than 80% of SNVs identified in FF specimens can be reliably recovered from the matched FFPE specimens. Furthermore, many studies found that a significantly higher number of unique SNVs can be identified from FFPE specimens than matched FF specimens and likely attributed to chemical modification of nucleotides by formalin-fixation. Specifically, formalin-fixation can cause deamination of cytosine bases to uracil. Thus, during amplification, if DNA polymerase reads across a uracil change, artefactual C>T/G>A changes can occur and introduce false positives [10]. Kerick et al. found that among the 149 false positives SNV calls from a FFPE specimen, all but four can be explained by the fixation process [88]. As an alternative, uracil-DNA glycosylase (UDG) was reported to be used to remove uracil-containing deaminated DNA molecules before library construction and treatment reduces C>T and A>G variant calls by 77% and 94%, respectively [93]. While FFPE specimens have a higher rate of nonreproducible SNVs, their random distributions allow for increased coverage to diminish the false positive rate [89]. One study showed that increasing sequencing coverage to 80x reduced significantly the false positive rate and increased the concordance between FF and FFPE specimens [88]. However, the depth of sequencing to produce reliable SNV calls is unrealistic for most whole genome sequencing and whole exome sequencing analysis.

Similar to SNV detection, FFPE specimens have also been evaluated for their feasibility for insertions and deletions (indel) detection. The concordance of indel calls between FFPE specimens and matched FF specimens has been mixed, ranging from 62% to 98.25% [88, 89, 91]. CNV estimations have also been inconsistent among studies with DNA-seq from FFPE specimens. Using whole genome sequencing, Schweiger et al. reported that the CNVs found were identical for FF and FFPE specimens [16]. However, Menon et al. used whole exome sequencing and reported that there is a high degree of noise in CNV calling from FFPE specimens, probably due to DNA degradation [15]. Munchel et al. used low-pass whole genome sequencing and found that the CNVs within segmented regions between paired FF and FFPE specimens are similar although the size of predicted CNVs differed between paired samples [89]. Several factors may have contributed to the relatively poor concordance of CNV calls between FF and FFPE specimens. First, FFPE specimens tend to have a high degree of cellular heterogeneity. A low purity of tumor cells or the presence of substantial immune cells can make CNV estimations noisy from FFPE specimens. Isolating pure population of tumor cells from FFPE specimen by flow cytometry based methods may circumvent this issue and improve CNV detection [87]. Another potential explanation for high CNV variation may stem from comparisons using lower coverage [89].

Together, these studies provide convincing evidence that accurate SNV can be identified from DNA-seq data from FFPE specimens and many studies have already taken advantage of large FFPE repository with DNA-seq technology to drive new scientific discoveries [93–103].

## 7. Applications in Other Type of HTS

DNA-seq has been modified to measure global DNA methylation patterns similar to methylation arrays using bisulfite treatment of DNA. Although less popular than DNA and RNA-seq, there have been successful usages of FFPE in bisulfite sequencing [104, 105]. One study evaluated the practicability of using FFPE specimens in bisulfite sequencing and found that the correlation between paired FFPE and FF specimens was good (*r* = 0.87) [106]. Several protocols and methodologies for bisulfite sequencing of FFPE specimens have been established [107, 108].

Chromatin immunoprecipitation sequencing (ChIP-seq) is a form of HTS that can identify global binding sites of DNA associated proteins. The usage of FFPE specimens for ChIP-seq can be difficult due to limited isolation of soluble DNA-protein complexes that are altered by excessive chemical cross-linking during formalin-fixation process. However, Fanelli et al. published a protocol, which demonstrated successful identification of DNA-protein binding sites using FFPE specimens [109]. This protocol has yet to be adapted widely for the usage of FFPE specimens. In 2016, Cejas et al. proposed a fixed-tissue chromatin immunoprecipitation sequencing (FiT-seq), which enables reliable extraction of soluble chromatin from FFPE specimens [110]. Whether this method will be more received by the research community remains to be seen. There are other types of HTS such as nuclear run-on assay (GRO-seq or PRO-seq) and cross-linking immunoprecipitation sequencing (CLIP-seq). These types of applications of HTS have not been used to the extent of DNA- and RNA-seq; thus few studies have been done using FFPE specimens.

## 8. NanoString

Similar to microarray technology, the NanoString nCounter system can directly measure gene expression by using multiplexed color-coded probe-pairs and offers high levels of precision and sensitivity (<1 copy per cell). The technology uses molecular “barcodes” and single molecule imaging to detect and count hundreds of unique transcripts in a single reaction. Because nCounter system is quantitative and does not require reverse transcription and amplification, it is free from any bias and errors introduced by the reverse transcription and the amplification processes. This is also the major reason for the claim that NanoString nCounter technology works well with FFPE specimens [111]. Naturally, several studies also investigated the performance of NanoString on FFPE specimens.

An original study conducted by NanoString company from 2008 measured concordance of gene expression measured by NanoString and RT-PCR/microarray and found high correlations (RT-PCR *R*^2^ = 0.79, Microarray *R*^2^ = 0.95). However, several additional follow-up studies found only moderate correlation between NanoString and RT-PCR, with correlation ranging from 0.48 to 0.59 [112–114]. This level of correlation holds true for both mRNA and miRNA measurement. In addition, the concordance of NanoString with other high throughput platforms, such as microarray and HTS, was also less than ideal, with correlations around 0.5 [14, 115–117]. On a positive note, NanoString was used with FFPE specimens to subtype diffuse large B-cell lymphoma [118]. The subtyping results by nCounter system have a 90% concordance rate with the results generated by Hans immunohistochemistry [118]. Based on the overall evidence presented thus far, we are not yet convinced that NanoString nCounter system is the definite technology for measuring gene expression from FFPE specimens. One of the major limitations of NanoString is that it is not a true high throughput technology, measuring up to a few hundred genes that have been chosen with prior knowledge. However, the limited throughput of NanoString is efficient enough to perform clinical assays such as Prosigna Panel and MammaPrint.

## 9. Discussion

FFPE processing of tissue is not the most ideal method for quantifying RNA and DNA variations with HTS methods. However, it is often chosen over FF storage because of minimal cost and ease of storage. With high throughput genomic assays dominating the biomedical research field, the ability to expand these studies to existing large FFPE specimen repositories can accelerate and rapidly verify discoveries. Numerous studies have been conducted to evaluate the performance of FFPE specimens with high throughput assays, including gene expression microarray, genotyping microarray, aCGH, methylation array, RNA-seq, DNA-seq, bisulfite sequencing, ChIP-seq, and NanoString. Together the current studies have established that FFPE can generate reliable data for gene expression and SNV detection. However, for more complex alterations such as indel, CNV estimation, and detection of hybrid transcripts, FFPE specimens have been proven to be less than ideal. The overall consensus for utilizing FFPE specimens in high throughput genomic study is that the data quality is negatively correlated to storage time. However, small RNAs have been shown to be an exception to this rule, due to the already small size of the small RNA which is less affected by the degradation of RNA.

Overall, FFPE specimens provide great value in biomedical research and can be utilized for HTS applications. However, there is always a high risk associated FFPE specimen based high throughput genomic assays because the quality of the FFPE specimens is near impossible to determine. Thus, a small pilot studies should be considered to establish feasibility prior to committing resources to a large FFPE based study.
